# Influence of Milk-Feeding Type and Genetic Risk of Developing Coeliac Disease on Intestinal Microbiota of Infants: The PROFICEL Study

**DOI:** 10.1371/journal.pone.0030791

**Published:** 2012-02-03

**Authors:** Giada De Palma, Amalia Capilla, Esther Nova, Gemma Castillejo, Vicente Varea, Tamara Pozo, José Antonio Garrote, Isabel Polanco, Ana López, Carmen Ribes-Koninckx, Ascensión Marcos, María Dolores García-Novo, Carmen Calvo, Luis Ortigosa, Luis Peña-Quintana, Francesc Palau, Yolanda Sanz

**Affiliations:** 1 Instituto de Agroquímica y Tecnología de Alimentos, Consejo Superior de Investigaciones Científicas (IATA-CSIC), Valencia, Spain; 2 Instituto de Biomedicina de Valencia (CSIC), CIBER de Enfermedades Raras (CIBERER), Valencia, Spain; 3 Department Metabolismo y Nutrición, ICTAN-CSIC, Madrid, Spain; 4 Unidad de Gastroenterología Pediátrica, Hospital Universitario Sant Joan de Reus, Tarragona, Spain; 5 Gastroenterología, Nutrición y Hepatología Pediátrica, Hospital Universitario Sant Joan de Deu and Unidad de Gastroenterología Pediátrica del Institut Dexeus, Barcelona, Spain; 6 Unidad de Gastroenterología Pediátrica, Hospital Clínico Universitario de Valladolid, Valladolid, Spain; 7 Servicio de Gastroenterología y Nutrición Pediátrica, Hospital Universitario La Paz, Madrid, Spain; 8 Unidad de Gastroenterología Pediátrica, Hospital Universitario La Fe, Valencia, Spain; 9 Unidad de Gastroenterología, Hospital Universitario Infantil Niño Jesús, Madrid, Spain; 10 Unidad de Gastroenterología, Hepatología y Nutrición Pediátrica, Hospital Universitario Nuestra Señora de Candelaria, Santa Cruz de Tenerife, Canarias, Spain; 11 Unidad de Gastroenterología, Hepatología y Nutrición Pediátrica, Hospital Universitario Materno-Infantil de Canarias, Universidad de Las Palmas de Gran Canaria, Las Palmas de Gran Canaria, Spain; Charité - Campus Benjamin Franklin, Germany

## Abstract

Interactions between environmental factors and predisposing genes could be involved in the development of coeliac disease (CD). This study has assessed whether milk-feeding type and HLA-genotype influence the intestinal microbiota composition of infants with a family history of CD. The study included 164 healthy newborns, with at least one first-degree relative with CD, classified according to their HLA-DQ genotype by PCR-SSP DQB1 and DQA1 typing. Faecal microbiota was analysed by quantitative PCR at 7 days, and at 1 and 4 months of age. Significant interactions between milk-feeding type and HLA-DQ genotype on bacterial numbers were not detected by applying a linear mixed-model analysis for repeated measures. In the whole population, breast-feeding promoted colonization of *C. leptum* group, *B. longum* and *B. breve*, while formula-feeding promoted that of *Bacteroides fragilis* group, *C. coccoides-E. rectale* group, *E. coli* and *B. lactis*. Moreover, increased numbers of *B. fragilis* group and *Staphylococcus* spp., and reduced numbers of *Bifidobacterium* spp. and *B. longum* were detected in infants with increased genetic risk of developing CD. Analyses within subgroups of either breast-fed or formula-fed infants indicated that in both cases increased risk of CD was associated with lower numbers of *B. longum* and/or *Bifidobacterium* spp. In addition, in breast-fed infants the increased genetic risk of developing CD was associated with increased *C. leptum* group numbers, while in formula-fed infants it was associated with increased *Staphylococcus* and *B. fragilis* group numbers. Overall, milk-feeding type in conjunction with HLA-DQ genotype play a role in establishing infants' gut microbiota; moreover, breast-feeding reduced the genotype-related differences in microbiota composition, which could partly explain the protective role attributed to breast milk in this disorder.

## Introduction

Celiac disease (CD) is a chronic inflammatory disorder of the small intestine that presents in genetically predisposed individuals following gluten consumption [1]. This disease often manifests in early childhood with small intestinal villous atrophy and signs of malabsorption [1]. Currently, a strict gluten-free diet is the only available treatment for patients but compliance with this dietary practice is extremely complex and, therefore, preventive strategies are being investigated [2].

The major genetic risk factor in CD is represented by Human Leukocyte Antigen (HLA)-DQ genes. Several studies have documented that the HLA-DQA1*05 and DQB1*02 alleles, encoding for particular DQ2 molecules, confer high susceptibility to CD [3]. This heterodimer can be encoded both in *cis* or *trans* forms. The susceptibility to CD is increased in homozygous subjects with a *cis* haplotype or possessing a second HLA-DQB1*02 allele [4]. In Europe, approximately 90% of patients have these genetic markers, whereas most of the remaining cases carry the HLA-DQA1*03 and DQB1*0302 alleles coding for DQ8 molecules [5]. Gluten is the main environmental factor responsible for the signs and symptoms of the disease but other environmental elements are also thought to play a role in the disease risk, including the type of milk-feeding, incidence of infections and intestinal dysbiosis [6]–[8].

Gut colonization starts immediately after birth and depends on multiple factors such as the type of delivery, contamination from the environment, the type of milk-feeding and, possibly, the genotype [9]–[11]. An adequate gut microbial colonization process contributes to the physiological development of the gut and the maturation of the immune system, thereby determining the risk of developing disease later in life. The early stage of colonization is characterized by the presence of higher levels of facultative anaerobes (enterobacteria, enterococci and streptococci) than of strictly anaerobic bacteria (e.g. bifidobacteria, bacteroides, clostridia, etc.); however, these proportions are reversed within a week following birth. In breast-fed infants *Bifidobacterium* spp. predominate, representing up to 90% of the total faecal microbiota, whereas in formula-fed infants the microbiota is more heterogeneous [9], [12], [13]. Breast-milk has been shown to be a continuous source of commensal bacteria to the infant gut, including species of the genera *Lactobacillus* and *Bifidobacterium* [14], [15]. It also contains prebiotic substances which are considered the main factors that stimulate the growth of *Bifidobacterium* spp. [16]. Epidemiological studies suggest that breast-feeding confers a protective effect against the risk of CD development, particularly when gluten is introduced in the diet while the infant is still breast-fed [1], [17]. However, the mechanisms underlying the beneficial effects of breast-milk on CD risk and their relationship with the gut microbiota are unknown. A preliminary study was previously conducted to test whether the HLA-DQ genotype could influence the composition of the gut microbiota, although the population under study was composed by a small number of exclusively breast-fed infants [18] and the aim was to establish the basis for this long-term study, including a representative cohort of infants.

The objective of this study was to assess the gut microbial colonization process during the first 4 months of life in breast-fed and formula-fed babies at risk of developing CD, by using quantitative PCR (qPCR). The ultimate purpose of our research is to gain a better understanding of the effects of early events leading to the acquisition of intestinal microbiota and their interactions with predisposing genes on CD risk.

## Methods

### Subjects and study design

A prospective observational study was carried out with a cohort of 164 healthy full-term newborns recruited between June 2006 and November 2010, who had a first-degree relative affected by CD. Data of mode of delivery, size, weight, weeks of gestation and type of feeding were recorded at birth and over the study period (Table 1). Infants were grouped for factors that influence the gut microbial colonization process, including age, genetic risk of CD development (HLA-DQ status), and type of milk-feeding (admitted randomly). The infants classified into the breast-fed group were those that received exclusively breast-feeding during the first 7 days of life, during the first month of life or during the first 4 months of life. Infants classified into the formula-fed group were those that received either exclusively formula or both formula and breast-milk at each sampling time. Infants were also grouped according the duration of breast-feeding (never breast-fed, breast-fed less than 1 month, breast-fed more than 1 month but less than 4 months and breast-fed for the 4 months). The study was approved by the ethics committees of Consejo Superior de Investigaciones Científicas (CSIC) and the Hospitals involved in the study, including Hospital Universitario Sant Joan de Reus, Hospital Universitario Sant Joan de Deu, Institut Dexeus, Hospital Clínico Universitario de Valladolid, Hospital Universitario La Paz, Hospital Infantil Universitario La Fe, Hospital Universitario Infantil Niño Jesús, Hospital Universitario Ntra Señora de Candelaria and Hospital Universitario Materno-Infantil de Canarias and conducted in accordance with the Helsinki Declaration of 1975 as revised in 1983. Written informed consent was obtained from the parents of infants included in the study.

**Table 1:** Demographic data of infants under study.

Demographic data		Total infants (n = 164)
**Delivery**		
	Vaginal	115/164
	Caesarean	49/164
1 **Size (cm)**		49.97 (2.42)
1 **Weight (g)**		3331.08 (537.49)
1 **Gestation (weeks)**		39.10 (1.44)
2 **Breast feeding**		
	7 days	115
	1 month	109
	4 months	64
3 **Formula feeding**		
	7 days	44
	1 month	55
	4 months	78
4 **Genetic risk of CD**		
	High genetic risk	48
	Intermediate genetic risk	69
	Low genetic risk	47

1 Data are expressed as mean and standard deviation in brackets.

2 Infants who were exclusively breast-fed at each sampling time were included in the breast-feeding group.

3 Infants who received either exclusively formula or both formula and breast-milk were included in the formula-feeding group.

4 Genetic risk of developing CD was established according to the HLA-DQ genotype (see Materials and Methods section for details).

### HLA-DQ Genotyping

DNA was extracted from yugal mucosa cells by scraping the inner side of the infants' cheek with sterile swabs (Copan innovation, Sarstedt, Germany) and purified according to the DNA IQ™ Casework Sample Kit for Maxwell® 16 protocol (Promega Biotech Iberica, Spain). Low-resolution HLA-DQB1 typing was performed by PCR-SSP (Polymerase Chain Reaction-Sequence Specific Primers) analysis [19]. Each PCR reaction was performed on about 20 ng of extracted DNA, 0.5 U of DNA polymerase (BIOTOOLS B&M S.A, Spain), 1× PCR Master Mix (Dynal *AllSet* ^+^™ SSP or *Olerup* SSP™) containing nucleotides (200 µmol L^−1^ each), PCR buffer, 5% glycerol and 100 µg mL^−1^ cresol red, 0·25 µmol L^−1^ of each allele- or group-specific primer pair and 0·1 µmol L^−1^ of internal positive control primer pair matching a segment of the human growth hormone gene in a final volume of 10 µL. An initial denaturation step at 94°C for 2 min was followed by 10 two-temperature cycles (94°C for 10 s and 65°C for 60 s) and 20 three-temperature cycles (94°C for 10 s, 61°C for 50 s and 72°C for 30 s). Detection of amplified alleles was carried out on 2% agarose gels after ethidium bromide staining. HLA-DQA1 alleles were genotyped in a stepwise fashion for high resolution typing to hone the risk classification of each individual.

### Faecal sampling and DNA extractions

Stool samples were collected from every subject at 7 days, 1 month and 4 months of age and frozen at −20°C immediately. Samples (1 g) were diluted 1∶10 (w/v) in PBS (pH 7.2) and homogenized by thorough agitation in a vortex. Aliquots were used for DNA extraction using the QIAamp DNA stool Mini kit (Qiagen, Hilden, Germany) following the manufacturer's instructions. DNA extractions from different pure cultures of reference strains were done following the same protocol.

### Quantitative PCR (qPCR) analysis of faecal bacteria

qPCR was used to quantify the different bacterial groups of the faecal microbiota using genus-, group- and species-specific primers as previously described [20], [21]. Briefly, PCR amplification and detection were performed with an ABI PRISM 7000-PCR sequence detection system (Applied Biosystems, UK) using SYBR® Green PCR Master Mix (SuperArray Bioscience Corporation, USA). The bacterial concentration from each sample was calculated by comparing the Ct values obtained from standard curves of reference strains. Standard curves were created using serial 10-fold dilutions of pure culture DNA corresponding to 10^2^ to 10^9^ cells, as determined by microscopy counts after staining with 4′, 6 diamino-2-phenylindole in an epifluorescence microscope (Olympus BX51, Tokio, Japan).

### Statistical analyses

Data were analysed using the SPSS 19.0 software for Windows (SPSS Inc, Chicago, IL, USA). The demographic characteristics of the study subjects are given as mean values (standard deviations [SDs]) for continuous variables and as numbers and proportions for categorical variables. Differences in demographic characteristic measures between the study groups were assessed using the Chi-Square Pearson test for categorical variables and ANOVA and *post hoc* LSD test for continuous variables. Microbiological data were transformed from exponential numbers into logarithms to adjust to normal and, in the tables, are expressed as mean values of log cells/g faeces and standard deviations (SDs). A mixed model with repeated measures with three fixed factors [genetic risk, type of milk-feeding and age (repeated measure)] was applied to determine the effects of genetic risk and type of feeding on bacterial counts. Interactions (magnitudinal) between these three factors were also analysed and not detected. Interactions between faecal microbial counts and type of delivery were not found and, therefore, data from infants with different type of delivery were grouped for statistical analyses. Differences between bacterial numbers at each sampling time (age) were analysed by ANOVA and *post hoc* LSD test. Correlations between factors (bacterial counts, age and genetic risk) were determined by Pearson correlation coefficients and correlations between bacterial counts and type of milk-feeding were tested by applying the Chi-Square Pearson test. In all cases, p-values less than 0.050 were considered statistically significant.

## Results

### Subjects and genetic risk of CD

The demographic characteristics of the infants under study are shown in Table 1. All newborns were full-term and most were delivered naturally (115 of 164). The size and weight of the infants at the moment of delivery were within standard parameters.

Infants were classified into three main groups according to their HLA-DQ genotype, and probabilities of developing CD were estimated according to previous studies [22], [23]. The first group included those individuals carrying the DQ2 haplotype in both *cis* (DQA1*0501-DQB1*0201 in homozygosis) and *trans* conformations (DQA1*0201-DQB1*0202 with DQA1*0505-DQB1*0301 in heterozygosis). The second group included those subjects carrying the DQ2 haplotype in *cis* conformation along with any other haplotype, as well as subjects carrying the DQ8 haplotype (DQA1*0301-DQB1*0302) in homozygosis. The third group included those individuals with other common genotypes not associated with CD. Of the 164 infants under study, 28.40% were classified in the first risk group, with the highest probability (>20%) of developing CD (the high genetic risk group) and 42.59% in the second group, with a >7% probability of developing CD (the intermediate genetic risk group). The remaining 29.01% of infants comprised the third group, with the lowest probability (<1%) of developing CD (the low genetic risk group).

### Influence of milk-feeding type on faecal microbiota of infants at risk of developing CD

The effect of milk-feeding type on the composition of the faecal microbiota, irrespectively of genotype, was evaluated over the study period. The type of milk feeding significantly influence different bacterial group counts by analysing data with the linear mixed model with time sampling as the repeated measure. *C. leptum* group (*P* = 0.005), *B. longum* (*P* = 0.050), and *B. breve* (*P* = 0.008) numbers were significantly higher in breast-fed than in formula-fed infants, whereas *Bacteroides fragilis* group (*P* = 0.004), *C. coccoides-E. rectale* group (*P*<0.001), *E. coli* (P = 0.026), and *B. lactis* (*P* = 0.002) numbers were higher in formula-fed infants than in breast-fed infants.

Correlations between milk-feeding type and several bacterial group counts at each sampling time were also analysed. Increased numbers of *C. leptum* group and *B. breve* correlated with breast-feeding at 7 days of age (r = −0.255, *P* = 0.012; r = −0.197, *P* = 0.036, respectively). Moreover, formula-feeding correlated with increased numbers of *E. coli*, *C. coccoides-E. rectal*e group and *B. lactis* at 1 month of age (r = 0.218, *P* = 0.006; r = 0.217, *P* = 0.011; r = 0.574, P = 0.002, respectively), and with increased numbers of *C. coccoides-E. rectal*e and *Bacteroides fragilis* groups at 4 months of age (r = 0.280, *P* = 0.001; r = 0.267, *P* = 0.004, respectively).

When analysing the cumulative effect of breast-feeding on the microbiota composition of 4-month-old infants, statistically significant negative correlations were established between increased *Bacteroides fragilis* and *C. coccoides-E. rectale* group counts and longer breast-feeding duration (r = −0.218, *P* = 0.020; r = −0.245, P = 0.003, respectively).

### Influence of HLA-DQ genotype on the fecal microbiota of infants at risk of developing CD

The influence of HLA-DQ genotype, irrespective of milk-feeding type, on fecal microbiota composition was established by applying a linear mixed-model analysis with sampling time as the repeated measure. According to this analysis, the effect of the genetic risk on bacterial numbers was significant for *Bifidobacterium* spp. (*P*<0.001) and *B. longum* (*P*<0.001), whose numbers increased when genetic risk of CD decreased. The effect of genetic risk was also significant for *Staphylococcus* spp. (*P* = 0.010) and *Bacteroides fragilis* group (*P* = 0.050), whose counts were higher when infants' genetic risk was greater. The influence of HLA-DQ genotype on fecal microbiota composition at each sampling time (7 days, 1 and 4 months) is also shown in Tables 2, 3 and 4.

**Table 2:** Faecal microbiota of infants with different HLA-DQ genotype at 7 days of age analysed by qPCR.

							2p-value		
	High risk		Intermediate risk		Low risk		High-	Intermediate-	High-
	n = 39		n = 67		n = 44		Intermediate	Low	Low
	1Mean	SD	Mean	SD	Mean	SD	Risk	Risk	Risk
***Bacteroides fragilis*** ** group**	4.54	1.59	4.32	1.34	4.12	1.49	0.538	0.623	0.352
***Staphylococcus*** ** spp.**	5.40	0.90	5.34	1.13	4.61	1.11	0.893	0.184	0.200
***C. coccoides - E. rectale*** ** group**	5.39	1.53	5.20	1.56	5.17	1.46	0.583	0.917	0.548
***C. leptum*** ** group**	4.36	1.21	4.44	1.47	4.23	1.54	0.841	0.563	0.749
***Lactobacillus*** ** group**	6.50	1.13	6.69	1.05	6.52	1.02	0.373	0.417	0.911
***E. coli***	6.19	1.93	5.71	1.93	6.00	1.85	0.258	0.505	0.710
***Bifidobacterium*** ** spp.**	6.38	1.79	6.99	1.66	7.43	1.42	0.087	0.200	0.007*
***B. longum***	5.27	1.29	6.12	1.58	6.21	1.72	0.012*	0.774	0.010*
***B. breve***	5.27	1.47	5.31	1.79	5.14	1.56	0.908	0.643	0.755
***B. bifidum***	4.47	0.97	4.67	1.45	4.89	1.27	0.523	0.432	0.206
***B. adolescentis***	4.74	0.79	4.90	1.50	5.23	0.45	0.788	0.575	0.410
***B. catenulatum***	4.69	1.38	5.41	1.61	5.11	1.85	0.193	0.523	0.487
***B. angulatum***	3.63	0.50	4.94	0.66	4.43	1.08	0.035*	0.260	0.184
***B. infantis***	5.62	0.58	5.78	1.91	4.80	0.69	0.819	0.215	0.300
***B. lactis***	3.91	0.48	3.89	0.55	4.22	0.63	0.959	0.244	0.229
***B. dentium***	4.42	0.22	4.27	0.61	4.21	-	-	-	-

1 Data are expressed as mean and standard deviation of log cells/g faeces.

2 Statistical significant differences were calculated using ANOVA and *post hoc* LSD test. Significant differences were established at p<0.050.

**Table 3:** Faecal microbiota of infants with different HLA-DQ genotype at 1 month of age analysed by qPCR.

							2p-value		
	High risk		Intermediate risk		Low risk		High-	Intermediate-	High-
	n = 48		n = 69		n = 47		Intermediate	Low	Low
	1Mean	SD	Mean	SD	Mean	SD	Risk	Risk	Risk
***Bacteroides fragilis*** ** group**	5.00	1.93	4.48	1.64	4.60	1.36	0.176	0.760	0.363
***Staphylococcus*** ** spp.**	6.12	1.09	5.17	1.01	4.66	0.93	0.014*	0.149	0.001*
***C. coccoides - E. rectale*** ** group**	5.75	1.20	5.55	1.70	5.53	1.36	0.517	0.950	0.512
***C. leptum*** ** group**	4.69	1.79	4.83	1.40	4.52	1.61	0.731	0.383	0.679
***Lactobacillus*** ** group**	6.72	1.08	6.93	1.04	6.79	0.97	0.291	0.475	0.746
***E. coli***	6.38	1.74	6.01	2.05	6.45	1.68	0.317	0.234	0.871
***Bifidobacterium*** ** spp.**	6.47	1.47	7.19	1.26	7.49	1.45	0.010*	0.282	0.001*
***B. longum***	5.55	1.67	6.11	1.58	6.44	1.63	0.001*	0.305	0.011*
***B. breve***	5.33	1.65	5.53	1.72	5.29	1.61	0.596	0.511	0.908
***B. bifidum***	4.98	1.38	4.78	1.53	4.77	1.29	0.503	0.958	0.510
***B. adolescentis***	5.48	1.14	4.71	0.68	5.44	1.37	0.140	0.122	0.936
***B. catenulatum***	4.90	1.59	5.31	1.74	4.69	1.34	0.344	0.139	0.663
***B. angulatum***	4.71	0.41	4.59	0.78	4.58	0.38	0.705	0.966	0.663
***B. infantis***	5.57	0.90	6.23	1.64	4.95	0.52	0.260	0.055	0.307
***B. lactis***	4.27	0.91	3.87	0.65	4.02	0.40	0.237	0.648	0.503
***B. dentium***	4.51	0.60	4.86	0.62	5.38	1.38	0.590	0.522	0.207

1 Data are expressed as mean and standard deviation of log cells/g faeces.

2 Statistical significant differences were calculated using ANOVA and *post hoc* LSD test. Significant differences were established at p<0.050.

**Table 4:** Faecal microbiota of infants with different HLA-DQ genotype at 4 months of age analysed by qPCR.

							2p-value		
	High risk		Intermediate risk		Low risk		High-	Intermediate-	High-
	n = 42		n = 65		n = 47		Intermediate	Low	Low
	1Mean	SD	Mean	SD	Mean	SD	Risk	Risk	Risk
***Bacteroides fragilis*** ** group**	5.52	2.09	4.88	1.86	4.91	1.60	0.137	0.932	0.188
***Staphylococcus*** ** spp.**	5.52	1.20	5.40	0.94	4.80	0.79	0.720	0.060	0.040*
***C. coccoides - E. rectale*** ** group**	6.00	1.43	6.04	1.55	5.88	1.26	0.898	0.590	0.716
***C. leptum*** ** group**	4.89	1.65	5.02	1.25	4.83	1.07	0.653	0.501	0.851
***Lactobacillus*** ** group**	6.70	0.98	6.99	0.94	6.95	0.92	0.117	0.816	0.210
***E. coli***	6.48	1.62	6.61	1.94	6.60	1.60	0.726	0.977	0.767
***Bifidobacterium*** ** spp.**	6.74	1.11	6.77	1.29	7.55	1.11	0.893	0.001*	0.002*
***B. longum***	6.06	1.40	6.22	1.32	6.76	1.31	0.570	0.040	0.019*
***B. breve***	5.99	1.73	5.94	1.66	5.98	1.62	0.886	0.901	0.982
***B. bifidum***	5.10	0.94	5.01	1.39	5.04	1.33	0.746	0.926	0.824
***B. adolescentis***	5.83	1.03	4.99	0.74	6.06	1.42	0.056	0.035*	0.648
***B. catenulatum***	5.65	1.67	5.42	1.74	5.57	1.32	0.608	0.722	0.866
***B. angulatum***	4.79	1.00	4.70	0.71	4.23	0.88	0.806	0.190	0.115
***B. infantis***	6.03	1.38	6.24	1.33	6.56	1.73	0.750	0.612	0.334
***B. lactis***	4.98	0.88	4.30	0.81	4.25	0.69	0.026	0.865	0.019*
***B. dentium***	4.54	0.48	4.37	0.71	4.74	-	-	-	-

1 Data are expressed as mean and standard deviation of log cells/g faeces.

2 Statistical significant differences were calculated using ANOVA and *post hoc* LSD test. Significant differences were established at p<0.050.

Correlations between genetic risk and several bacterial group counts at different infant ages (sampling time) were also established. Statistically significant correlations were found between increased numbers of both *Bifidobacterium* spp. (r = 0.235, *P* = 0.007 at 7 days; r = 0.268, *P* = 0.001 at 1 month; r = 0.257, *P* = 0.001 at 4 months) and *B. longum* (r = 0.209, *P* = 0.013 at 7 days; r = 0.207, *P* = 0.011 at 1 month; r = 0.200, *P* = 0.016 at 4 months) and reduced genetic risk during the whole sampling period. In contrast, increased numbers of *Staphylococcus* spp. at 1 and 4 months (r = −0.465, *P* = 0.001 at 1 month; r = −0.278, *P* = 0.038 at 4 months) and *B. lactis* at 4 months (r = −0.332, *P* = 0.026) correlated with increased genetic risk.

### Influence of the HLA-DQ genotype in the faecal microbiota of either breast-fed or formula fed infants

The microbiota composition of infants grouped according to milk-feeding type was also analysed as a function of HLA-DQ genotype over the study period to eliminate the effects of milk-feeding type. In breast-fed infants, the effect of genetic risk of CD was significant on bacterial counts of *Bifidobacterium* spp. (*P* = 0.046), which decreased when the genetic risk was increased, and on bacterial counts of *Staphylococcus* spp. (*P* = 0.030), *C. leptum* group (*P* = 0.047), *B. adolescentis* ((*P* = 0.028) and *B. dentium* (*P* = 0.009), which increased when the genetic risk was also increased, according to linear mixed-model analysis with sampling time (age) as the repeated measure. The differences in mean bacterial numbers according to the genetic risk of developing CD in breast-fed infants analysed at each sampling point are also shown in Tables 5, 6 and 7.

**Table 5:** Faecal microbiota of breast-fed infants with different HLA-DQ genotype at 7 days of age analysed by qPCR.

							2p-value		
	High risk		Intermediate risk		Low risk		High-	Intermediate-	High-
	n = 27		n = 50		n = 31		Intermediate	Low	Low
	1Mean	SD	Mean	SD	Mean	SD	Risk	Risk	Risk
***Bacteroides fragilis*** ** group**	4.19	1.58	4.43	1.32	3.99	1.42	0.570	0.378	0.710
***Staphylococcus*** ** spp.**	5.40	0.94	5.51	1.01	4.94	1.19	0.826	0.460	0.580
***C. coccoides - E. rectale*** ** group**	5.28	1.67	5.19	1.62	4.98	1.29	0.819	0.625	0.514
***C. leptum*** ** group**	4.38	1.30	4.70	1.49	4.38	1.36	0.471	0.418	0.995
***Lactobacillus*** ** group**	6.55	1.26	6.64	1.07	6.41	0.94	0.727	0.360	0.632
***E. coli***	6.03	1.93	5.54	1.79	6.18	1.80	0.315	0.199	0.780
***Bifidobacterium*** ** spp.**	6.61	1.89	7.14	1.64	7.52	1.51	0.224	0.362	0.057
***B. longum***	5.49	1.31	6.18	1.66	6.42	1.83	0.098	0.522	0.039*
***B. breve***	5.44	1.41	5.81	1.78	5.07	1.69	0.430	0.106	0.447
***B. bifidum***	4.52	1.00	4.88	1.54	4.91	1.39	0.334	0.920	0.324
***B. adolescentis***	5.36	0.64	5.14	1.54	5.16	0.54	0.792	0.972	0.824
***B. catenulatum***	4.77	1.63	5.72	1.69	5.54	2.20	0.212	0.787	0.358
***B. angulatum***	3.40	0.42	4.92	0.59	4.27	1.08	0.048*	0.207	0.229
***B. infantis***	5.59	0.64	5.80	2.13	5.23	0.04	0.827	0.656	0.778
***B. lactis***	3.81	0.54	4.02	0.59	4.07	0.68	0.535	0.890	0.464
***B. dentium***	4.42	0.22	4.35	0.65	-	-	-	-	-

1 Data are expressed as mean and standard deviation of log cells/g faeces.

2 Statistical significant differences were calculated using ANOVA and *post hoc* LSD test. Significant differences were established at p<0.050.

**Table 6:** Faecal microbiota of breast-fed infants with different HLA-DQ genotype at 1 month of age analysed by qPCR.

							2p-value		
	High risk		Intermediate risk		Low risk		High-	Intermediate-	High-
	n = 28		n = 53		n = 30		Intermediate	Low	Low
	1Mean	SD	Mean	SD	Mean	SD	Risk	Risk	Risk
***Bacteroides fragilis*** ** group**	4.87	1.72	4.56	1.67	4.87	1.37	0.480	0.497	0.988
***Staphylococcus*** ** spp.**	5.59	0.82	5.16	0.97	4.64	1.03	0.430	0.223	0.121
***C. coccoides - E. rectale*** ** group**	5.57	1.41	5.37	1.75	5.22	1.44	0.636	0.717	0.461
***C. leptum*** ** group**	4.81	2.21	4.99	1.48	4.69	1.43	0.735	0.483	0.824
***Lactobacillus*** ** group**	6.66	1.18	6.85	1.08	6.73	1.05	0.449	0.625	0.804
***E. coli***	6.39	1.49	5.56	1.93	6.52	1.66	0.064	0.030	0.792
***Bifidobacterium*** ** spp.**	6.74	1.57	7.20	1.32	7.45	1.40	0.200	0.468	0.077
***B. longum***	5.83	1.96	5.95	1.63	6.24	1.52	0.768	0.477	0.373
***B. breve***	5.55	1.82	5.29	1.71	5.26	1.73	0.594	0.952	0.580
***B. bifidum***	5.34	1.41	4.51	1.34	4.72	1.41	0.030*	0.550	0.135
***B. adolescentis***	5.68	1.35	4.64	0.76	5.23	1.11	0.122	0.272	0.493
***B. catenulatum***	5.15	1.97	5.11	1.88	4.81	1.64	0.953	0.644	0.660
***B. angulatum***	4.69	0.55	4.76	0.94	4.52	0.45	0.900	0.637	0.700
***B. infantis***	5.58	0.96	6.55	1.55	5.40	0.19	0.156	0.257	0.848
***B. lactis***	3.87	0.30	3.46	0.42	3.96	0.40	0.254	0.064	0.769
***B. dentium***	4.14	0.14	4.86	0.62	6.35	-	-	-	-

1 Data are expressed as mean and standard deviation of log cells/g faeces.

2 Statistical significant differences were calculated using ANOVA and *post hoc* LSD test. Significant differences were established at p<0.050.

**Table 7:** Faecal microbiota of breast-fed infants with different HLA-DQ genotype at 4 months of age analysed by qPCR.

							2p-value		
	High risk		Intermediate risk		Low risk		High-	Intermediate-	High-
	n = 11		n = 39		n = 21		Intermediate	Low	Low
	1Mean	SD	Mean	SD	Mean	SD	Risk	Risk	Risk
***Bacteroides fragilis*** ** group**	4.47	1.93	4.77	1.77	4.51	1.67	0.636	0.640	0.956
***Staphylococcus*** ** spp.**	4.52	0.33	5.42	0.90	4.27	0.51	0.102	0.041	0.712
***C. coccoides - E. rectale*** ** group**	5.81	1.84	5.47	1.35	5.45	1.09	0.485	0.969	0.502
***C. leptum*** ** group**	5.70	1.28	4.90	1.09	4.08	0.76	0.075	0.020*	0.002*
***Lactobacillus*** ** group**	6.51	1.00	6.92	0.90	6.96	0.88	0.190	0.876	0.188
***E. coli***	6.15	1.46	6.43	1.90	6.32	1.71	0.662	**0.832**	0.814
***Bifidobacterium*** ** spp.**	7.23	1.22	6.80	1.32	7.33	1.29	0.347	0.156	0.833
***B. longum***	6.54	1.38	6.25	1.29	6.37	1.32	0.532	0.741	0.740
***B. breve***	6.46	2.48	5.95	1.71	6.14	1.65	0.508	0.691	0.691
***B. bifidum***	5.41	1.31	4.95	1.38	4.96	1.24	0.335	0.971	0.384
***B. adolescentis***	6.68	1.83	4.71	0.55	6.70	0.94	0.015	0.006	0.975
***B. catenulatum***	5.49	1.60	5.07	1.76	5.38	0.93	0.591	0.598	0.897
***B. angulatum***	4.46	1.15	4.33	0.69	4.30	-	-	-	-
***B. infantis***	4.92	-	6.54	1.77	6.89	1.88	-	-	-
***B. lactis***	4.09	0.23	4.12	0.85	4.00	0.66	0.953	0.819	0.883
***B. dentium***	4.29	-	4.14	0.66	-	-	-	-	-

1 Data are expressed as mean and standard deviation of log cells/g faeces.

2 Statistical significant differences were calculated using ANOVA and *post hoc* LSD test. Significant differences were established at p<0.050.

Correlations between genetic risk and bacterial counts were also analysed and these were significant between increased genetic risk of CD development and increased *C. leptum* group counts in breast-fed infants (r = −0.408, *P* = 0.004).

In formula-fed infants, the effect of genetic risk on *Bifidobacterium* spp. and *B. longum* counts was found significant (*P*<0.001 and *P*<0.001, respectively) by applying a linear mixed model analysis with sampling time as the repeated measure, and those infants with reduced genetic risk had increased numbers of these bacterial groups. The effect of genetic risk on numbers of *B. fragilis* group and *Staphylococcus* spp. was also significant, following the opposite trend (*P* = 0.008 and *P* = 0.004, respectively). The differences in mean bacterial numbers according to the genetic risk of developing CD in formula-fed infants analysed at each sampling point are also shown in Tables 8, 9 and 10.

**Table 8:** Faecal microbiota of formula-fed infants with different HLA-DQ genotype at 7 days of age analysed by qPCR.

							2p-value		
	High risk		Intermediate risk		Low risk		High-	Intermediate-	High-
	n = 11		n = 17		n = 13		Intermediate	Low	Low
	1Mean	SD	Mean	SD	Mean	SD	Risk	Risk	Risk
***Bacteroides fragilis*** ** group**	5.39	1.34	3.79	1.37	4.06	1.63	0.046*	0.723	0.103
***Staphylococcus*** ** spp.**	5.40	1.04	4.82	1.46	4.40	1.27	0.560	0.673	0.377
***C. coccoides - E. rectale*** ** group**	5.65	1.19	5.25	1.46	5.55	1.84	0.541	0.640	0.890
***C. leptum*** ** group**	4.32	1.08	3.59	1.02	3.44	2.37	0.290	0.852	0.314
***Lactobacillus*** ** group**	6.37	0.74	6.84	1.01	6.82	1.20	0.241	0.973	0.280
***E. coli***	6.60	1.97	6.11	2.25	5.47	2.05	0.578	0.513	0.303
***Bifidobacterium*** ** spp.**	5.73	1.35	6.61	1.72	7.23	1.23	0.164	0.287	0.029*
***B. longum***	4.81	1.17	5.95	1.39	5.79	1.32	0.031*	0.758	0.087
***B. breve***	4.79	1.63	4.38	1.45	5.32	1.20	0.515	0.125	0.450
***B. bifidum***	4.28	0.92	4.13	1.03	4.84	0.94	0.763	0.090	0.308
***B. adolescentis***	4.12	0.12	3.70	-	5.37	0.27	0.692	0.431	0.808
***B. catenulatum***	4.53	0.83	4.77	1.24	4.37	0.68	-	-	-
***B. angulatum***	4.09	-	5.01	1.14	5.42	-	-	-	-
***B. infantis***	5.74	-	5.69	-	4.06	0.08	-	-	-
***B. lactis***	4.15	0.14	3.52	0.07	4.48	0.54	0.109	0.032*	0.317
***B. dentium***	-	-	3.88	-	4.21	-	-	-	-

1 Data are expressed as mean and standard deviation of log cells/g faeces.

2 Statistical significant differences were calculated using ANOVA and *post hoc* LSD test. Significant differences were established at p<0.050.

**Table 9:** Faecal microbiota of formula-fed infants with different HLA-DQ genotype at 1 month of age analysed by qPCR.

							2p-value		
	High risk		Intermediate risk		Low risk		High-	Intermediate-	High-
	n = 18		n = 16		n = 17		Intermediate	Low	Low
	1Mean	SD	Mean	SD	Mean	SD	Risk	Risk	Risk
***Bacteroides fragilis*** ** group**	5.28	2.42	4.46	1.70	3.94	1.18	0.317	0.537	0.126
***Staphylococcus*** ** spp.**	6.43	1.16	5.23	1.20	4.70	0.86	0.069	0.446	0.018*
***C. coccoides - E. rectale*** ** group**	6.00	0.84	6.02	1.59	6.12	0.99	0.958	0.822	0.780
***C. leptum*** ** group**	4.57	1.28	4.37	1.15	4.20	1.96	0.751	0.774	0.554
***Lactobacillus*** ** group**	6.81	0.92	7.10	0.99	6.91	0.86	0.376	0.569	0.753
***E. coli***	6.38	2.11	7.66	1.55	6.20	1.74	0.053	0.031*	0.788
***Bifidobacterium*** ** spp.**	6.06	1.24	7.39	0.75	7.73	1.40	0.002*	0.400	<0.001*
***B. longum***	5.14	1.04	6.68	1.30	6.87	1.82	0.003*	0.701	0.001*
***B. breve***	5.05	1.41	5.89	1.52	5.34	1.39	0.126	0.338	0.590
***B. bifidum***	4.53	1.24	5.62	1.83	4.85	1.09	**0.041**	0.145	0.544
***B. adolescentis***	5.23	1.01	4.84	0.55	6.28	2.53	0.698	0.231	0.391
***B. catenulatum***	4.62	1.08	5.70	1.43	4.57	1.00	0.041*	0.025*	0.922
***B. angulatum***	4.74	0.32	4.51	0.84	4.73	0.03	0.644	0.696	0.986
***B. infantis***	5.51	-	4.32	-	4.64	0.57	-	-	-
***B. lactis***	4.43	1.05	4.45	0.29	4.38	-	-	-	-
***B. dentium***	5.25	0.30	-	-	4.40	-	-	-	-

1 Data are expressed as mean and standard deviation of log cells/g faeces.

2 Statistical significant differences were calculated using ANOVA and *post hoc* LSD test. Significant differences were established at p<0.050.

**Table 10:** Faecal microbiota of formula-fed infants with different HLA-DQ genotype at 4 months of age analysed by qPCR.

							2p-value		
	High risk		Intermediate risk		Low risk		High-	Intermediate-	High-
	n = 30		n = 26		n = 27		Intermediate	Low	Low
	1Mean	SD	Mean	SD	Mean	SD	Risk	Risk	Risk
***Bacteroides fragilis*** ** group**	6.05	2.00	5.13	2.01	5.30	1.48	0.132	0.791	0.218
***Staphylococcus*** ** spp.**	5.75	1.22	5.38	1.05	4.91	0.81	0.410	0.301	0.043*
***C. coccoides - E. rectale*** ** group**	6.06	1.28	6.81	1.50	6.20	1.30	0.049*	0.112	0.718
***C. leptum*** ** group**	4.66	1.69	5.18	1.43	5.31	0.96	0.214	0.762	0.122
***Lactobacillus*** **group**	6.76	0.98	7.08	1.01	6.94	0.96	0.227	0.594	0.510
***E. coli***	6.61	1.69	7.00	1.92	6.79	1.52	0.421	0.659	0.715
***Bifidobacterium*** ** spp.**	6.56	1.03	6.81	1.18	7.71	0.95	0.364	0.003*	<0.001*
***B. longum***	5.89	1.39	6.15	1.41	7.04	1.25	0.500	0.022*	0.003*
***B. breve***	5.88	1.54	5.92	1.61	5.86	1.62	0.930	0.905	0.969
***B. bifidum***	4.98	0.75	5.09	1.44	5.10	1.42	0.752	0.981	0.737
***B. adolescentis***	5.64	0.83	5.45	0.82	5.57	1.65	0.751	0.865	0.916
***B. catenulatum***	5.71	1.74	5.84	1.68	5.72	1.58	0.826	0.849	0.980
***B. angulatum***	4.89	1.00	5.22	0.33	4.23	0.92	0.489	0.044*	0.093
***B. infantis***	6.11	1.40	6.00	1.03	6.32	1.66	0.884	0.690	0.735
***B. lactis***	5.28	0.80	4.39	0.82	4.31	0.71	0.016*	0.785	0.007*
***B. dentium***	4.56	0.50	4.85	0.72	4.74	-	-	-	-

1 Data are expressed as mean and standard deviation of log cells/g faeces.

2 Statistical significant differences were calculated using ANOVA and *post hoc* LSD test. Significant differences were established at p<0.050.

Statistically significant correlations were also established between the genetic risk of CD development and bacterial group numbers in formula-fed infants at different sampling times. At 7 days, reduced numbers of *Bifidobacterium* spp. correlated with increased genetic risk of developing CD (r = 0.362, *P* = 0.028). At 1 month, reduced numbers of *Bifidobacterium* spp. and *B. longum* correlated with increased genetic risk of developing CD (r = 0.511, *P*<0.001 and r = 0.454, *P* = 0.001, respectively), and increased numbers of *Staphylococcus* spp. correlated with increased genetic risk of developing CD (r = −0.573, *P* = 0.013). At 4 months, reduced numbers of *Bifidobacterium* spp. and *B. longum* correlated with increased genetic risk of developing CD (r = 0.412, *P*<0.001 and r = 0.336, *P* = 0.002, respectively), and increased numbers of *Staphylococcus* spp. and *B. lactis* correlated with increased genetic risk (r = −0.345, *P* = 0.040 and r = −0.442, *P* = 0.010, respectively).

The cumulative effect of breast-feeding on microbiota composition was also evaluated at 4 months of age and some significant correlations were established. When infants had never been breast-fed, significant correlations between increased genetic risk and low numbers of *Bifidobacterium* spp., and *B. longum* were detected (r = 0.343, P = 0.026; r = 0.455, P = 0.003, respectively). Similarly, when infants were breast-fed for more than 1 month and less than 4 months, low numbers of *Bifidobacterium* spp. correlated with high genetic CD risk (r = 0.408, P = 0.031), whereas high *B. lactis* numbers correlated with high genetic CD risk (r = −0.697, P = 0.006). On the other hand, when breast-feeding was exclusive until the fourth month of age, the high genetic risk correlated with increased numbers of *C. leptum* group (r = −0.454, P = 0.001).

## Discussion

This is the first report on the effects of both milk feeding type and HLA-DQ genotype on the gut microbial colonization process of healthy full-term infants with a family history of CD. In the present study, milk-feeding type influenced the composition of the microbiota, partially in agreement with previous studies [24]. Breast-feeding favoured *C. leptum* group, *B. longum* and *B. breve* gut colonization, while formula-feeding favoured that of *B. lactis*, *E. coli*, *C. coccoides-E. rectale* group and *B. fragilis* group. Similar trends were detected when considering the cumulative effect of breast-feeding particularly for *C. coccoides-E. rectale* and *B. fragilis* groups.

In addition, specific features of fecal microbiota were associated with the genetic risk of developing CD, based on HLA-DQ genotype, when considered irrespectively of milk-feeding type. Increased numbers of *Bifidobacterium* spp. and *B. longum* were characteristic of microbiota of infants with the lowest genetic risk, whereas increased numbers of *Staphylococcus* spp. and *B. fragilis* group were characteristic of that of infants with the highest genetic CD risk. To date, there is limited evidence of a correlation between genotype and intestinal microbiota composition in humans. In previous human studies, monozygotic twins were demonstrated to have more similar faecal bacterial DNA profiles than unrelated individuals [25] and monozygotic twins more so than dizygotic twins [10]. More recently a strong bond between genotype, phenotype and changes in gut microbiota has been reported in patients with Cohn's disease, demonstrating that specific gene alterations (e.g. NOD2 and ATG16L1) can have an impact on intestinal microbiota composition [26]. Animal and human studies indicate that the host genotype may influence factors such as the repertoire of mucins, which act as bacterial adhesion sites in the intestinal mucosa, as well as the immune responses. Together, these can contribute to modulating the colonization of certain microorganisms [27]. In this context, our results suggest that HLA-DQ genotype influences the microbial colonization pattern early in life and, therefore, could be an additional factor influencing the risk of developing CD later in life. Enterocytes, which are in close proximity with intestinal content and bacteria, can express HLA class II molecules of the MHC to a certain extent and are able to act as antigen presenting cells [28]. Moreover, HLA class II molecules are primarily expressed by dendritic cells present in the lamina propria that can sample the mucosal surface for microbial antigens, which can be presented to naïve B and T cells after processed to peptides that are loaded on MHC class I and class II molecules. This is a critical step in triggering the mucosal innate immune response, which could restrict bacterial colonization and influence disease risk [29]. In particular, the increased *Staphylococcus* counts detected in the high CD genetic risk group of infants could favour the activation of a robust T-cell response by the preferential interaction of certain superantigens, such as staphylococcal superantigen A, with HLA-DQ molecules, thereby enhancing the risk of T-cell mediated diseases [30], such as CD.

The influence of the HLA-DQ genotype was also analysed in subgroups of either breast-fed or formula fed infants to gather more information about the respective effects of each variable (genotype and milk-feeding type) on the microbiota of the infants under study. In the whole infant population, formula feeding favoured the presence of increased numbers of *B. fragilis* group, which were also higher in infants with higher genetic risk of developing CD in the whole population and in the subgroup of formula-fed infants, suggesting that the colonization of this bacterial group is greatly defined by type of milk-feeding. Nevertheless, increased counts of *Staphylococcus* spp. were associated with increased genetic risk of developing CD in the whole population and in both breast- and formula-fed infant subgroups, but their colonization was not favoured by formula feeding, suggesting that the HLA-DQ genotype plays a more prominent role in the colonization of this bacterial group. Notably, reduced numbers of *Bifidobacterium* spp. were associated with an increased risk of developing CD in the whole population and in both breast- and formula-fed infants, and the colonization of species of this genus was also favoured by breast-feeding. Therefore, the findings suggest that bifidobacterial numbers can be influenced by both the HLA-DQ genotype and the milk-feeding type. Moreover, low counts of the species *B. longum* were found in infants of higher risk to develop CD in the whole population and in the subgroup of formula-fed infants but not in the subgroup of breast-fed infants indicating that the breast-feeding is providing to the infant's gut microbiota certain *Bifidobacterium* spp. [31], [16], which could partially explain the protective role attributed to breast feeding in the risk of developing CD in previous epidemiological studies [17].

These and other commensal bacteria are recognized as constituting major *stimuli* for the adequate development of immune functions and oral tolerance [6], which could also be related to the risk of developing CD. In another prospective study, a reduced ratio of *Bifidobacterium* to *Clostridium* counts in the faecal microbiota of infants was shown to precede the development of atopic diseases later in life, indicating that the relative proportions of these bacterial groups may favour or protect against the development of immune-related disorders [32]. Moreover, *Bifidobacterium* spp. and *B. longum* levels in both biopsies and faeces have been reported lower in CD patients than in healthy controls [31], [33]. However, breast feeding also favoured the presence of increased numbers of *C. leptum* group when this factor was considered alone, and this bacterial group was associated with an increased risk of developing CD in the subgroup of breast-fed infants. *C. leptum* group numbers were also reported to be higher in the fecal and duodenal microbiota of CD patients than in healthy controls [34].

Overall, this study demonstrates that the milk-feeding type and the HLA-DQ genotype differently influence the bacterial colonization pattern of the newborn intestine during the first 4 months of life and, therefore, could also influence the risk of developing CD in later life. Breast-feeding reduced the genotype-related differences in microbiota composition, which could partly explain the protective role attributed to breast milk in this disorder. Further studies are underway to reveal additional evidence of the role played by early intestinal colonization patterns in CD development in this cohort of infants.
